# First case of nodular dermatofibrosis with cystic renal disease in a beagle: An atypical case without FLCN gene mutation

**DOI:** 10.1002/vms3.1401

**Published:** 2024-03-07

**Authors:** Belén Rivera Gómez‐Barris, Veronica Balazs, Katherine Chaguay Villamar, Constanza Toro‐Valdivieso

**Affiliations:** ^1^ Oftaderm: Oftalmología y Dermatología Veterinaria Santiago Chile; ^2^ Dermatopatía: Dermatologa Veterinaria Práctica Privada Ecuador; ^3^ Department of Veterinary Medicine University of Cambridge Cambridge UK

**Keywords:** beagle, canine, nodular dermatofibrosis, renal cysts

## Abstract

This report presents the first case of nodular dermatofibrosis with renal cysts (NDRC) in a beagle. In this atypical case, the gene mutation associated with the disease was not present, the renal cysts showed dynamic changes in size and number, and the patient has greatly surpassed the NDRC life expectation.

## Graphical abstract

We report the first case of nodular dermatofibrosis with cystic renal (NDRC) disease in a beagle dog. Contrary to our expectations, the patient did not carry the genetic mutation associated with this presentation. Although these cases have a poor prognosis, the patient has greatly surpassed the NDRC life expectation.

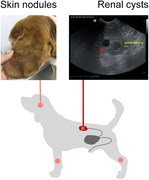

## 1 BACKGROUND

Nodular dermatofibrosis (ND) was first described in German shepherd dogs (GS), the most predisposed breed (Suter et al., 1983). The syndrome is often associated with a gene mutation and is characterised by a progressive appearance of multiple cutaneous and subcutaneous nodules of firm consistency. The nodules consist primarily of dense collagen fibres and are mostly distributed around the limbs and head. The size of the nodules may vary between a few millimetres to several centimetres.

Although the skin nodules are present in all the cases, ND reports are often accompanied with renal cysts, cystadenomas or cysteadenocarcinomas (NDRC) (Moe & Lium, 1997; Suter et al., 1983). The renal lesions may appear uni‐ or bilaterally although it seems that the compromise of both kidneys is related to the progression of the illness. In female dogs, the pathology is often presented with uterine tumours as well (Moe & Lium, 1997). In most cases, patients are first presented at the veterinary practice due to the skin nodules but otherwise they seem healthy. In these early cases, renal lesions often become evident months to a couple years after the patient was first presented with the skin lesions. Cases of NDRC have a poor prognosis and patients often die or are euthanised within 2 years since the renal neoplasia is first identified due to the progression of the renal cysts or the evolution of the skin nodules, which can lead to limb deformities(2, 4–8). Patients with unilateral or no renal lesions tend to survive for longer after first diagnosis (Ciccarelli et al., 2019; Zanatta et al., 2013).

The etiology of NDRC remains poorly understood. Nonetheless, there is compelling evidence that shows that the syndrome has a genetic predisposition. More specifically, a genetic study in GS dogs identified an autosomal dominant mutation in the exon 7 of the folliculin gene (FCLN) (Lingaas et al., 2003). The association between this mutation and NDRC appears to be consistent in GS; however, it is not yet clear if this is the case in non‐GS dogs. There are only two cases where the genetic test has been performed in non‐GS dogs, one of which did not show the mutation (Ciccarelli et al., 2019; Zanatta et al., 2013).

Almost all the published cases of NDRC have been reported in GS and both females and males can be affected. There are few reports on other breeds such as in a golden retriever, boxer, cross breed dogs as well as one report on an Australian cattle dog (Gardiner & Spraker, 2008; Marks et al., 1993; White & Schultheiss, 1998). Here we present, to the best of our knowledge, the first report of NDRC in a beagle dog.

## 2 CASE PRESENTATION

### 2.1 Anamnesis

An 8‐year‐old intact male beagle dog was presented with several skin nodules. The nodules had grown slowly over 2 years, first in the extremities and later on the external portion of the ears and head.

The patient's previous veterinarian had prescribed depot corticosteroids (Triamcinolone, 0.2 mg/kg single dose) and weekly baths using regular shampoo. There was no sign of improvement after a month of treatment.

### 2.2 Clinical examination

**FIGURE 1:**
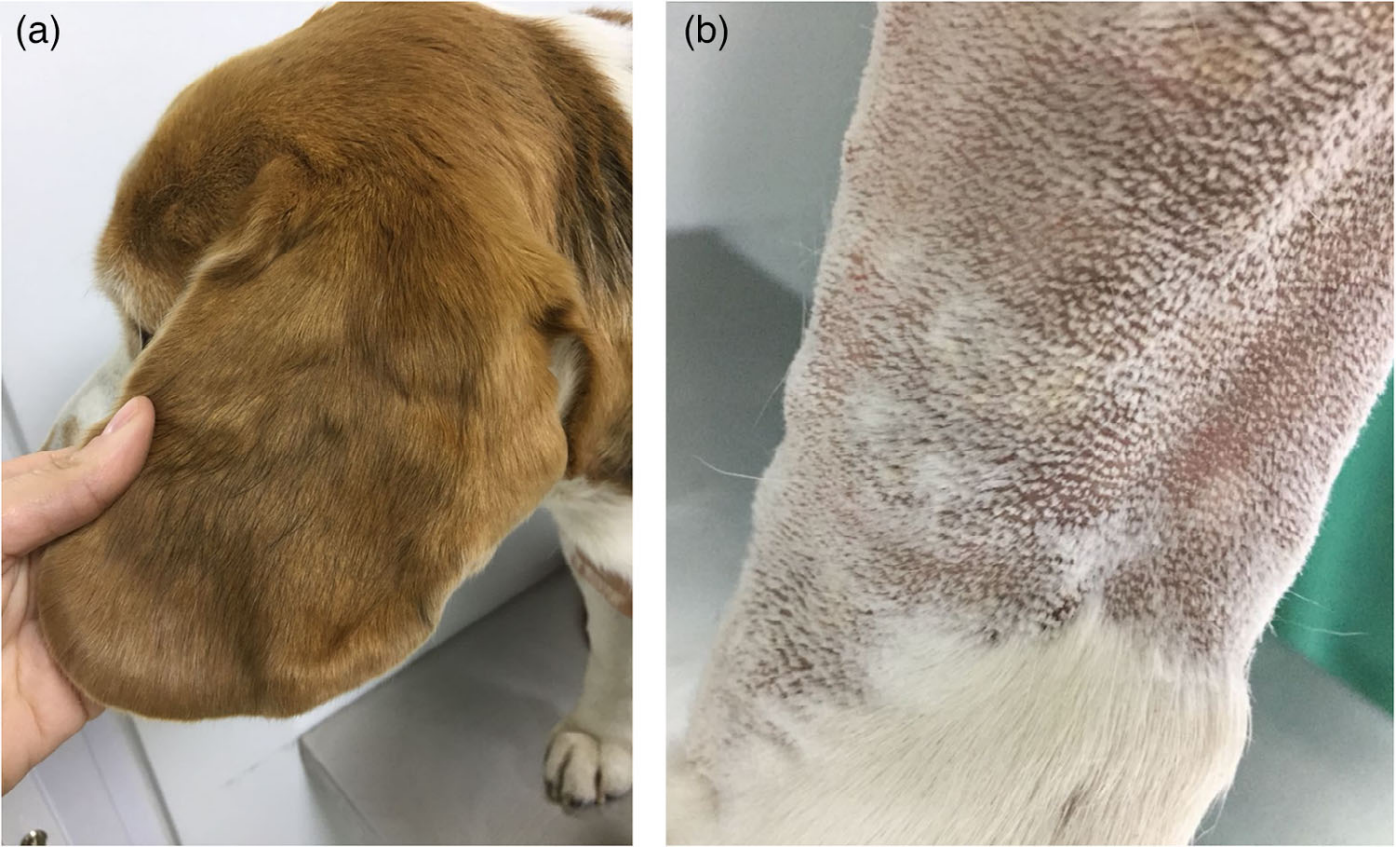
Multiple skin nodules in ears (a) and limbs (b).

During the clinical examination, numerous well‐circumscribed skin nodules of firm consistency were found in all four extremities, pinna and head (more than 15 in each location). The nodule sizes varied between 0.5 cm and 4 cm, approximately. There were no ulcerations, pruritus or other signs of discomfort due to the skin lesions (Figure 1).

A fine‐needle aspiration biopsy of the skin nodules was collected and analysed during consultation. Fibroblasts were the predominant cell type present in the sample. An additional sample was collected and sent for further cytological examination. The analysis revealed moderate cellularity and slight haemodilution with cells of intermediate size with varying shapes from oval to spindle with mild to moderate anisocytosis and anisocariosis, and a moderate to high nucleus‐cytoplasmic ratio. The cytoplasm was basophilic with poorly defined borders and a round to oval nucleus with fine chromatin. Mature adipose tissue was observed on one of the slides. These findings were indicative of a mesenchymal neoplasm with adipose tissue.

For a more accurate diagnostic approximation, three tissue samples were collected and sent for histopathology. The samples showed elevated structures covered with hyperkeratotic epidermis, located in the superficial and deep dermis. The findings were identified as neoplastic processes characterised by fibroblasts. Spindle cells organised in bundles with abundant collagen fibres and sparse extracellular matrix was found beneath the epithelium. The histopathological findings were suggestive of fibromas.

No evident alterations in the haemogram and serum biochemistry analysis were found. However, the hormonal profile revealed an alteration in thyroxine (T4, value: < 0.5 µg/dL; ref. values: 1.3–3.8 µg/dL) and thyroid‐stimulating hormone (TSH, value: 0.04 µg/mL; ref. values: 0.1–0.5 µg/mL). Therefore, the patient was diagnosed with hypothyroidism.

**FIGURE 2:**
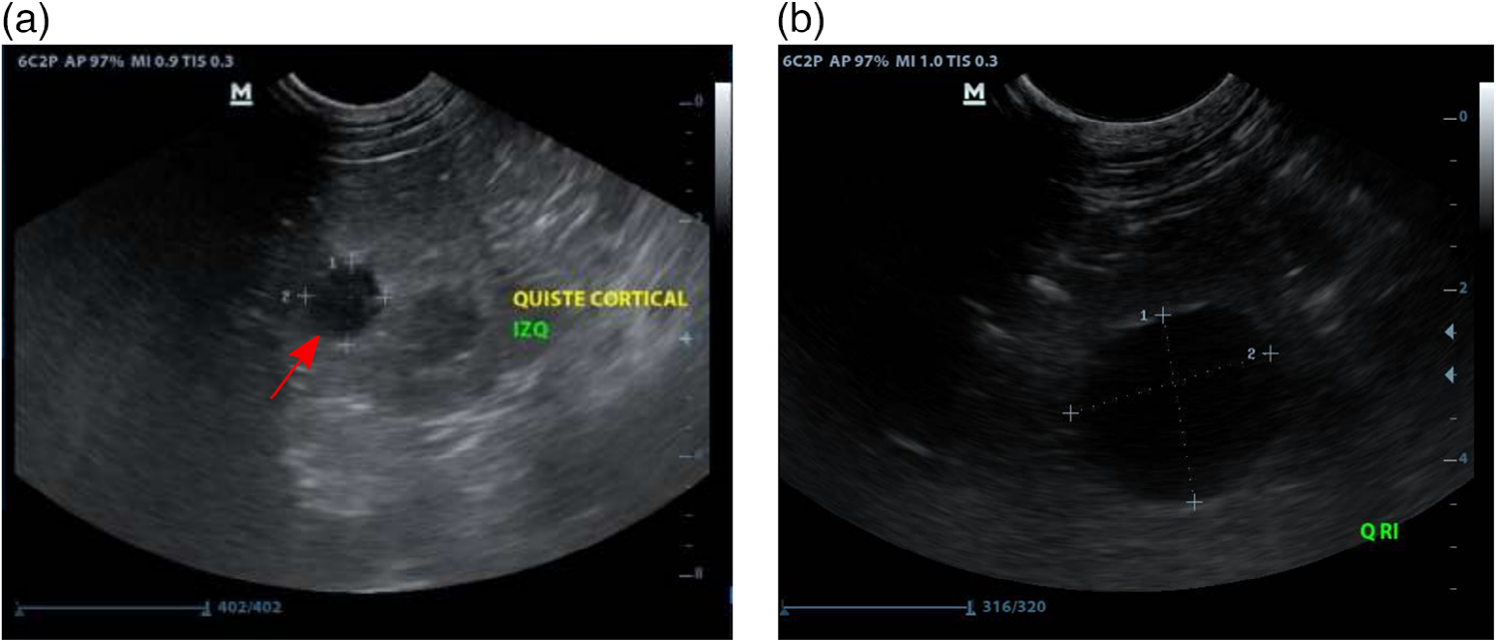
Ultrasound examination of the left kidney. (a) Red arrow shows a 1.41×1.37 cm. (b) Ultrasound of the left kidney taken 14 months later. Red arrow in (b) shows a 2.66 cm × 2.59 cm cyst.

An ultrasound scan showed a 1.5 × 0.45 cm cortical cyst in the left kidney. In a follow‐up exam, 6 months later, a 0.22 cm cyst was found in the right kidney. A year after the first scan the cyst found in the left kidney had more than doubled its size (2.66 cm × 2.59 cm). The time‐lapse ultrasound scans showed bilateral cortical renal cysts suggesting a proliferative process (Figure 2).

Finally, we conducted a polymerase chain reaction (PCR) to look at the FLCN gene but there was no evidence of the gene mutation associated with NDRC (Lingaas et al., 2003). Despite the negative PCR, the symptoms were consistent with previous reports of NDRC. The PCR was conducted at the Department of Veterinary Medicine, University of Cambridge, UK.

### 2.3 Treatment and progression

Seven years since the first ultrasound the patient is still alive and his hypothyroidism is under control. There is no curative treatment for NDRC. The management of the syndrome is mostly palliative when necessary. Additionally, the patient's hypothyroidism and NDRC development are closely monitored once every 6 months where urinary track ultrasound and blood tests (complete blood count and serum biochemistry) are performed. Interestingly, instead of the renal cysts following a linear progressive development, here, regular ultrasound examinations show that the cysts are following a more dynamic progression with occasional remissions which has not been described before. Similarly, blood tests do not show further abnormalities. The patient has greatly surpassed the life expectation of the NDRC syndrome.

## 3 DISCUSSION

This report presents an atypical case of NDRC in a beagle dog, the first case ever reported in this breed. The dynamic size changes in the cysts found in both kidneys observed in this case are especially interesting as it has not been previously described. The disease typically has a very poor prognosis, where patients die within 2 years after renal cysts are first identified. The unexpectedly prolonged survival of the patient and regular ultrasonographic monitoring may have allowed the observation of these temporal variations in the cysts' attributes. Whether these dynamic changes are part of the normal course of NDRC or unusual phenomena remains unclear. We encourage other practitioners to conduct regular urinary ultrasounds to see if these temporal variations are common. If these changes are characteristic of NDRC, understanding their triggers may impact our knowledge of their progression and help develop better supportive treatment.

In contrast to GS cases, we did not find the FLCN mutation. While the FLCN mutation has shown to be consistent in NDRC cases in GS, its involvement in other breeds is unclear. Unfortunately, the molecular analysis needed to assess this mutation is rarely performed in a clinical setting. Moreover, there are only three published cases where non‐GS dogs have been tested for the mutation (Ciccarelli et al., 2019; Zanatta et al., 2013), including the case presented here. Only one of them, a mixed‐breed dog, presented the mutation. The absence of the dog's family history prevents ruling out a possible GS influence in the patient's genetic lineage. Meanwhile, the other two cases do not support the link between the specific FLCN gene mutation identified by Lingaas et al. (2003) and NDRC in non‐GS. To investigate the relationship between the FLCN mutation and NDRC in non‐GS dogs, it is vital that practitioners perform the PCR test. However, unknown mutations in other related genes may exist.

Overall, more studies in non‐GS NDRC cases are needed to gain a deeper insight into the pathology, causes and predispositions of the syndrome in this group. However, finding cases in other breeds is unusual. Finally, this case report contributes to NDRC awareness and promotes the clinical preparedness of practitioners receiving similar cases.

## AUTHOR CONTRIBUTIONS

Belén Rivera: Data curation, formal analysis, funding acquisition, investigation, methodology, project administration, original draft, review & editing. Veronica Balazs: Formal analysis, investigation. Katherine Chaguay Villamar: Data curation, investigation. Constanza Toro‐Valdivieso: Formal analysis, original draft, review & editing.

## CONFLICT OF INTEREST STATEMENT

The authors declare that they have no conflict of interest.

### ETHICAL STATEMENT

Not applicable

### PEER REVIEW

The peer review history for this article is available at https://www.webofscience.com/api/gateway/wos/peer-review/10.1002/vms3.1401.

## Data Availability

Data sharing is not applicable to this article as no new data were created or analysed in this study.
